# Evaluation of the Toxicity on Lung Cells of By-Products Present in Naphthalene Secondary Organic Aerosols

**DOI:** 10.3390/life11040319

**Published:** 2021-04-06

**Authors:** Yuri Lima de Albuquerque, Emmanuelle Berger, Sophie Tomaz, Christian George, Alain Géloën

**Affiliations:** 1 UMR Ecologie Microbienne, Université Claude Bernard Lyon 1, 69622 Villeurbanne, France; yuri-mateus.lima-de-albuquerque@univ-lyon1.fr (Y.L.d.A.); emmanuelle.danty@univ-lyon1.fr (E.B.); 2 Univ Lyon, Université Claude Bernard Lyon 1, 69100 Villeurbanne, France; sophie.tomaz@inrs.fr (S.T.); christian.george@ircelyon.univ-lyon1.fr (C.G.)

**Keywords:** PAHs, secondary organic aerosols, RTCA, Holomonitor, naphthoquinone, A549

## Abstract

In 2018, seven million people died prematurely due to exposure to pollution. Polycyclic aromatic hydrocarbons (PAHs) are a significant source of secondary organic aerosol (SOA) in urban areas. We investigated the toxic effects of by-products of naphthalene SOA on lung cells. These by-products were 1,4-naphthoquinone (1,4-NQ), 2-hydroxy-1,4-naphthoquinone (2-OH-NQ), phthalic acid (PA) and phthaldialdehyde (OPA). Two different assessment methodologies were used to monitor the toxic effects: real-time cell analysis (RTCA) and the Holomonitor, a quantitative phase contrast microscope. The chemicals were tested in concentrations of 12.5 to 100 µM for 1,4-NQ and 1 to 10 mM for 2-OH-NQ, PA and OPA. We found that 1,4-NQ is toxic to cells from 25 to 100 µM (EC50: 38.7 µM ± 5.2); 2-OH-NQ is toxic from 1 to 10mM (EC50: 5.3 mM ± 0.6); PA is toxic from 5 to 10 mM (EC50: 5.2 mM ± 0.3) and OPA is toxic from 2.5 to 10 mM (EC50: 4.2 mM ± 0.5). Only 1,4-NQ and OPA affected cell parameters (migration, motility, motility speed and optical volume). Furthermore, 1,4-NQ is the most toxic by-product of naphthalene, with an EC50 value that was one hundred times higher than those of the other compounds. RTCA and Holomonitor analysis showed a complementarity when studying the toxicity induced by chemicals.

## 1. Introduction

In 2010, the World Health Organization (WHO) expressed concerns regarding the effects of several air pollutants on health [1]. In 2018, the WHO estimated that 7 million people suffered premature death caused by exposure to fine particles in polluted air. These particles can penetrate deep into the lungs and cardiovascular systems, causing diseases including stroke, heart disease, lung cancer, chronic obstructive pulmonary diseases and respiratory infections, including pneumonia [2,3].

Naphthalene, a ubiquitous polycyclic aromatic hydrocarbon (PAH), is one of the several air pollutants that affect the human health [1]. Naphthalene is a semi-volatile organic compound (SVOC) according to the U.S. Environmental Protection Agency classification [4], and it has a series of anthropogenic sources, such as chemical industries, gasoline evaporation or oil burning, leading locally to acute exposures [1,5]. In urban areas, vehicle emissions represent the most important source, but naphthalene is also present in the smoke of cigarettes [6]. Once in the air, naphthalene undergoes atmospheric oxidation, mainly by reacting hydroxyl radicals (•OH), and thereby acts as a precursor of secondary organic aerosols (SOA). These ultrafine particles have a complex chemical composition and therefore possess chemical complexity and yield products such as 1,4-naphthoquinone (1,4-NQ), 2-hydroxy-1,4 naphthoquinone (2-OH-NQ), phthalic acid (PA), phthaldialdehyde (OPA) [7,8,9,10].

Studies on lung cell lines showed that 1,4-naphthoquinone (1,4-NQ) is toxic [11]. Quinones are a class of organic compounds with chemical properties allowing them to interact with biological targets by forming covalent bonds and transferring electrons in oxidation–reduction reactions. Among all quinones, 1,4-NQ and its secondary products are of particular interest because of their occurrence as natural chemicals and/or products [12,13]. These compounds have diverse pharmacological properties, such as antimicrobial [14], antiviral [15], antiprotozoal and anthelmintic [16,17], also presenting cytotoxic effects on cancer cell lines [18]. Moreover, 2-hydroxy-1,4-naphthoquinone (lawsone) (2-OH-NQ), a photochemical reaction product of 1,4-naphthoquinones, can also be extracted from the leaves of *Lawsonia inermis.* It is one of the most widely used skin dyes, commonly known as Henna. It has been intensively utilized for many years in the biology, medicine, agriculture and industries as a skin dye and antifungal [19,20]. Regarding its toxicity, Sauriasari et al. concluded that lawsone is not mutagenic, but it is toxic to cells in a dose-dependent manner [21].

Phthalic acid (PA) is a benzene dicarboxylic acid with no reports on its toxic effects on the respiratory system [22]. Phthaldialdehyde (OPA) is a dialdehyde used mostly as a fluorescent marker or a disinfectant, but in vivo experiments proved OPA to be toxic to the respiratory systems of rats [23,24,25,26].

To study the cytotoxic effects of chemicals, the colorimetric MTT (tetrazolium salt (3-[4,5-dimethylthiazol-2-yl]-2,5-diphenyltetrazolium bromide)) assay, is widely used. However, this technique only shows the end-points and not the stage of cell proliferation [27]. Nowadays, more sophisticated approaches are available. Real-time cell analysis (RTCA) allows the label-free and real-time monitoring of cell proliferation and viability. It monitors the viability of cultured cells using electrical impedance. The continuous monitoring of cell proliferation makes it possible to recognize different perturbations of cell viability, such as toxicity (cell death) and reduced proliferation (cell cycle arrest). Technical improvements also allow the creation of 3D reconstructive images of cells and the measurement of the structural and behavioral parameters of cells [28,29]. Similarly, the Holomonitor microscope performs label-free and non-invasive measurements with the use of a low-power laser. Cell structural (area, optical volume and perimeter) and behavioral (motility, motility speed and migration) parameters can be measured in real time with this microscope.

Bearing in mind the importance of ultrafine particles derived from PAH photo-oxidation in urban areas, we studied here the toxicity of the main by-products of naphthalene SOA using the abovementioned new methodologies to determine their impacts on lung cells.

## 2. Materials and Methods

### 2.1. Cell Culture

Human carcinoma A549 cells (provided by ATCC, Massanas, VA, USA) were grown in Dulbecco’s Modified Eagle’s Medium with low glucose (1 g/L) containing 10% fetal calf serum (DMEM 10% FCS, PAA Laboratories, Toronto, ON, Canada) with the addition of streptomycin plus penicillin (100 units/mL; Sigma-Aldrich, St. Quentin Fallavier, France). The cells were trypsinized by 0.05% trypsin (Sigma-Aldrich, St. Quentin Fallavier, France) and the cell concentration was measured using a Sceptor pipette (Millipore, Burlington, MA, USA). Cell cultures were conducted from passage 3 to 7.

### 2.2. Naphthalene SOA By-Products

The chemical composition of PAH-derived SOA is quite complex, with hundreds to thousands of compounds being present. To simplify our approach, and to allow a molecular approach to the toxic response of these particles, we investigated the impact of by-products mimicking naphthalene-derived aerosols [7,8,9,10,30].

The chemicals investigated in this study were 1,4-naphthoquinone (1,4-NQ), 2-hydroxy-1,4 naphthoquinone (2-OH-NQ), phthalic acid (PA) and phthaldialdehyde (OPA), purchased from Sigma-Aldrich (St. Quentin Fallavier, France). All chemicals were dissolved in DMSO and further diluted in Dulbecco’s Modified Eagle’s Medium with low glucose (1 g/L) containing 10% fetal calf serum (DMEM 10% FCS, PAA Laboratories, Toronto, ON, Canada) with the addition of streptomycin plus penicillin (100 units/mL; Sigma Aldrich, St-Louis, MO, USA) with 0.1% of DMSO (Sigma Aldrich, St-Louis, MO,USA) in the end.

Cells were exposed to different concentrations of the compounds when the cell index reached the value of 1.0. The concentrations tested for 1,4-NQ were 12.5, 25, 50 and 100 µM. The concentrations for 2-OH-NQ, phthalic acid (PA) and phthaldialdehyde (OPA) were 1, 2.5, 5, 7.5 and 10 mM.

### 2.3. Measurements of Cytotoxicity 

#### 2.3.1. Effects on Cell Proliferation

Real-time cell analysis system (RTCA) (Agilent, Santa Clara, CA, USA) measures cell indexes resulting from cell surface occupancy, taking into consideration cell number, cell size and adhesion force. In the case of cytotoxicity, a decrease in cell index can result from a decreased cell number (=cell death), a decrease in cell adhesion or a decrease in cell surface. Cell surface and number can be measured by the Holomonitor. RTCA results are represented as delta cell indexes (differences in final − initial cell index = time of treatment). In order to measure the cytotoxic response of A549 cells in real time, cells were seeded on gold microelectrodes embedded at the bottom of 96-well microplates (E-plates; Roche Diagnostics, Basel, Switzerland) at a density of 2500 cells/well. The impedance was recorded at 15-min intervals for 24 h in a standard 37 °C cell culture incubator with 5% CO_2_. The EC50 of each compound was calculated using the RTCA data with Excel.

#### 2.3.2. Quantitative Phase Imaging

Quantitative phase imaging was performed using the Holomonitor M4 digital holographic cytometer (DHC) from Phase Holographic Imaging (PHI, Lund, Sweden). The platform was housed in a standard 37 °C cell culture incubator with 5% CO_2_. The behavioral (motility, motility speed and migration) and structural (area, optical volume and perimeter) parameters were measured from 3D reconstructed images obtained for 10 h with intervals of 5 min between measurements.

### 2.4. Statistical Analysis

Data presented are representative of experiments performed at least in triplicate, as mean values ± SEM for RTCA and Holomonitor (n = 3 for 96- and n = 8 for E96 wells). Statistical analysis was performed with StatView 4.5 software (Abacus Corporation, Baltimore, MD, USA) for Windows. Data were analyzed using one-way ANOVA followed by Fisher’s protected least significance difference (PLSD) post hoc test. Significance level was accepted at *p* < 0.05.

## 3. Results

### 3.1. Effects of Naphthalene SOA By-Products on A549 Proliferation

To determine whether the by-products of naphthalene SOA are toxic, A549 cells were exposed to increasing concentrations of 1,4-NQ (12.5, 25, 50 and 100 µM), 2-OH-NQ, PA and OPA (1, 2.5, 5, 7.5 and 10 mM).

**Figure 1:**
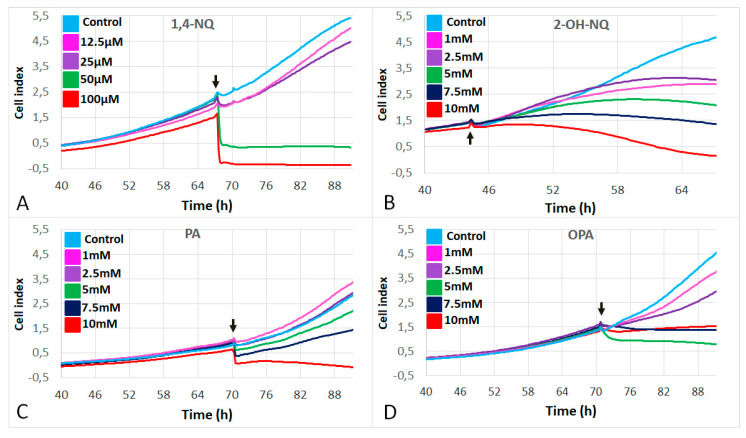
Dose–response curves of A549 cell proliferation in response to (**A**) 1,4-naphthoquinone (1,4-NQ) at 12.5 µM (pink), 25 µM (purple), 50 µM (green), 100 µM (red) and control (light blue); (**B**) 2-hydroxy-1,4-naphthoquinone (2-OH-NQ); (**C**) phthalic acid (PA); and (**D**) phthaldialdehyde (OPA). For (**B**–**D**): 1 mM (pink); 2.m (purple); 5 mM (green); 7.5 mM (blue); and 10 mM (red) and control (light blue). Black arrows: time of addition of the treatment (TRT).

Figure 1 shows the evolution of cell index given by RTCA of A549 cells in response to increasing concentrations of each by-product of naphthalene. We found that 1,4-NQ killed the cells at concentrations of 50 and 100 µM, while at 25 µM, it promoted toxicity by decreasing the cell index. At 10 µM, 1,4-NQ did not show any sign of toxicity, thus not differing from the control (Figure 1A). Furthermore, 2-OH-NQ was toxic to cells at every tested concentration, promoting cell death at 10mM and decreasing the cell index at 1, 2.5, 5 and 7.5 mM (Figure 1B). PA killed the cells at 10 mM. At 5 and 7.5 mM, it caused a decrease in cell index, while at 1 and 2.5 mM, no differences from the control were observed (Figure 1C). OPA was toxic from 2.5 to 10 mM, significantly decreasing the cell index. At the concentration of 1 mM, it was not toxic to cells (Figure 1D).

**Figure 2:**
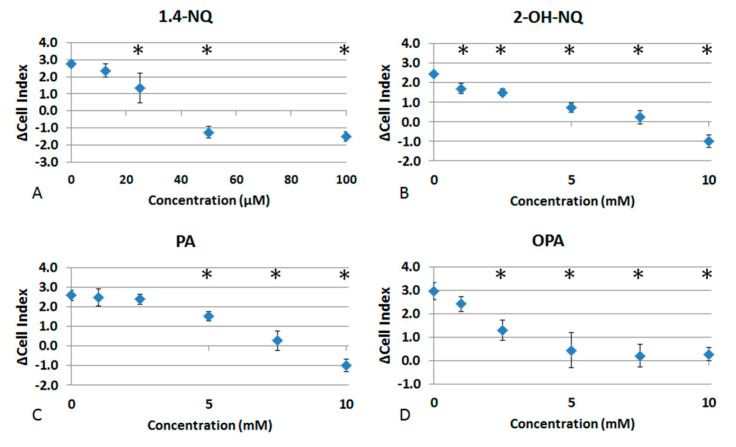
Delta cell index in response to increasing concentrations of (**A**) 1,4-naphthoquinone (1,4-NQ); (**B**) 2-hydroxy, 1,4-naphthoquinone (2-OH-NQ); (**C**) phthalic acid (PA); and (**D**) phthaldialdehyde (OPA). Asterisks indicate significant differences at *p* < 0.05 when compared to control.

Figure 2 represents the EC50 for each chemical. They were calculated from the mean of the delta cell indexes obtained from at least three repetitions. Results are 38.7 ± 5.2 µM for 1,4-NQ; 5.3 ± 0.6 mM for 2-OH-NQ; 5.2 ± 0.3 mM for PA; and 4.2 ± 0.5 mM for OPA.

### 3.2. Effects of Naphthalene SOA By-Products on A549 Behavioral and Structural Parameters

To determine whether the by-products of naphthalene SOA alter the structure and behavior of cells, A549 cells were exposed to concentrations corresponding to the EC50 observed for cell proliferation, i.e., 1,4-NQ (50 µM), 2-OH-NQ, PA and OPA (5 mM). Cells were monitored by means of the Holomonitor for 10 hours post-exposure to the chemicals.

**Figure 3:**
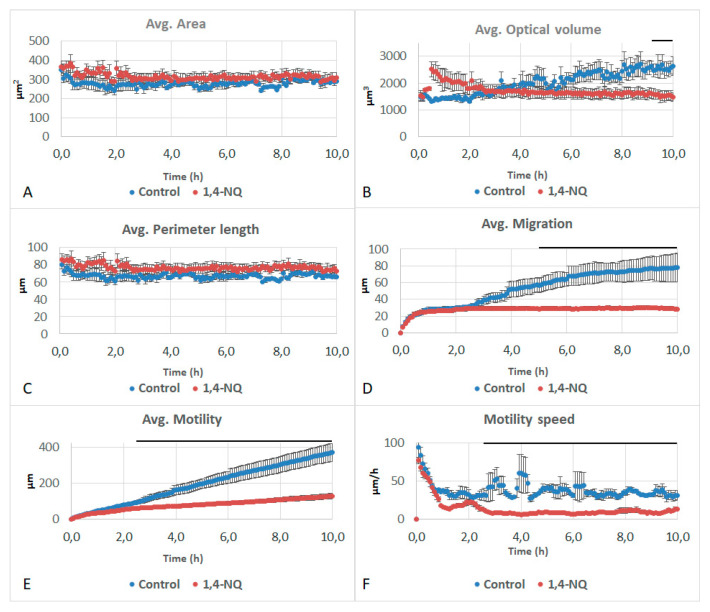
Effects of 50 µM of 1,4-naphthoquinone (1,4-NQ) on (**A**) area, (**B**) optical volume, (**C**) perimeter length, (**D**) migration, (**E**) motility and (**F**) motility speed of A549 cells. Black lines above the curves indicate significant differences at *p* < 0.05 between 1,4-NQ and control.

Firstly, 1,4-NQ impacted the optical volume, migration, motility and motility speed of the cells, while their area and perimeter did not differ from the control. Optical volume showed a significant decrease when compared to the control in the last 2 h of the experiment (Figure 3A–C). Migration of cells treated with 1,4-NQ showed a significant decrease when compared to the control after 5 h, as did motility and motility speed after 3 h (Figure 3D–F). The curve for migration, differing from the control, did not evolve during the experiment. Motility, however, slightly increased throughout the experiment but remained much lower than the control.

**Figure 4:**
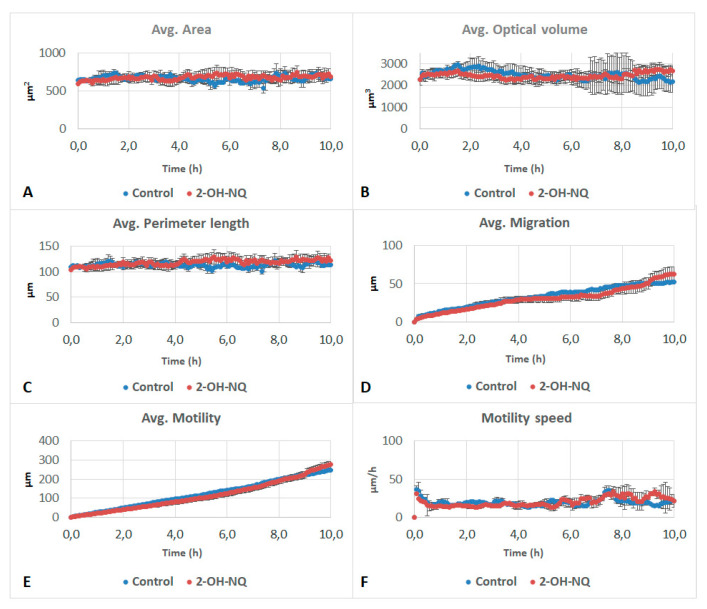
Effects of 5 mM of 2-hydroxy-1,4-naphthoquinone (2-OH-NQ) on (**A**) area, (**B**) optical volume, (**C**) perimeter length, (**D**) migration, (**E**) motility and (**F**) motility speed of A549 cells. No significant differences were observed in these parameters.

**Figure 5:**
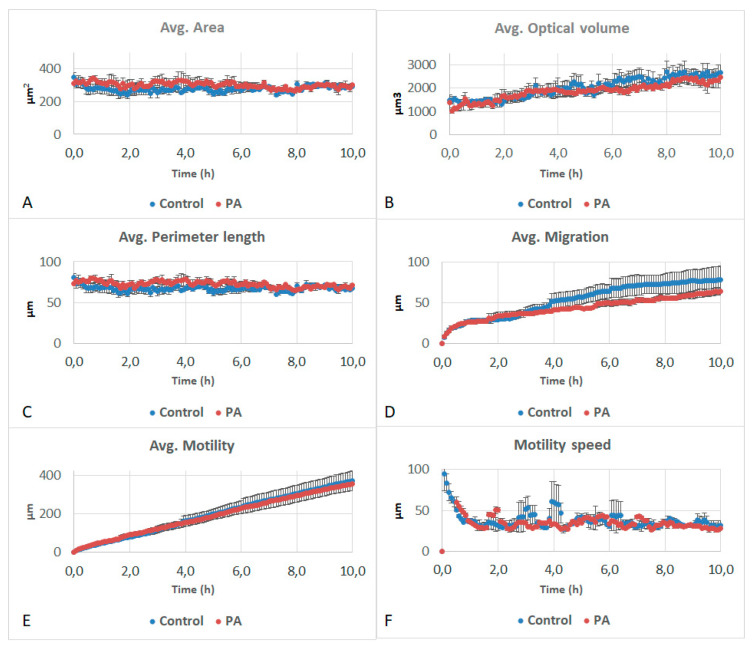
Effects of 5 mM of phthalic acid (PA) on the (**A**) area, (**B**) optical volume, (**C**) perimeter length, (**D**) migration, (**E**) motility and (**F**) motility speed of A549 cells. No significant differences were observed in these parameters.

**Figure 6:**
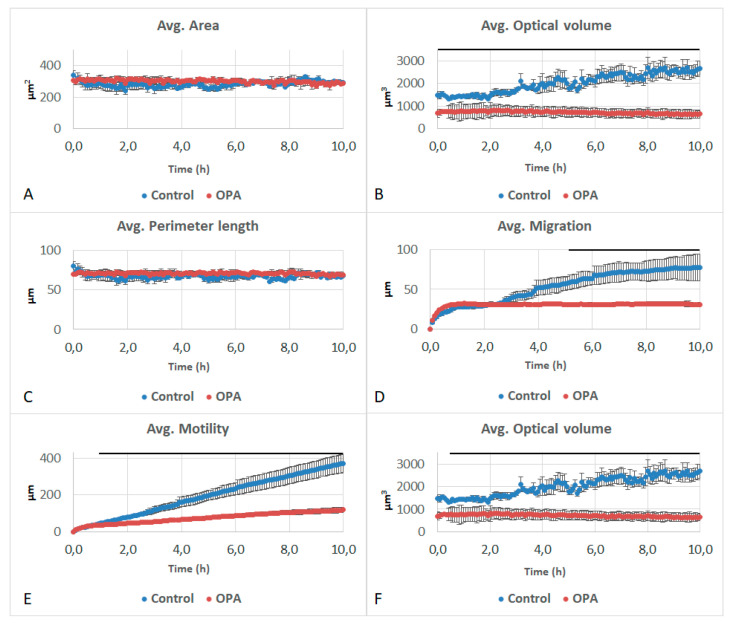
Effects of 5 mM of phthaldialdehyde (OPA) on (**A**) area, (**B**) optical volume, (**C**) perimeter length, (**D**) migration, (**E**) motility and (**F**) motility speed of A549 cells. Black lines above the curves represent significant differences at *p* < 0.05 between OPA and the control.

Furthermore, 2-OH-NQ and PA did not affect any of the studied parameters (Figure 4 and Figure 5). OPA impacted the optical volume, migration, motility and motility speed of the cells. While area and perimeter did not differ from the control (Figure 6A,C), optical volume showed a significant reduction over the entire experiment, when compared to the control (Figure 6B). After 5 h, migration showed a significant decrease, as did motility after 1 h and motility speed after 45 min (Figure 6D–F). Optical volume and migration remained constant throughout the experiment. Motility increased slightly during the experiment, but at a slower pace than the control.

## 4. Discussion

Although air pollution is a threat to human health, there are still major uncertainties regarding the nature of the compounds triggering health diseases. In particular, in populated urban areas, exposure to secondary aerosols arising from the photo-oxidation of PAHs is significant. In the present study, the toxicity of four chemicals derived from naphthalene SOA was measured using an RTCA biosensor. The dose–response curves show that 1,4NQ is by far the most toxic (EC50 = 38.7 µM) compared to 2-OH-NQ, PA and OPA (EC50 = 5.3, 5.2 and 4.2 mM, respectively), by an order of magnitude of 100. Babich & Stern reported that substitution with a hydroxyl group in the quinoid ring of 1,4-NQ, thus creating 2-OH-NQ, occasioned a decrease in its cytotoxicity, corroborating the present results [31]. Shang et al. found that 1,4-NQ had a dose–response toxic effect on A549 cells from 2.5 to 50 µM [32]. While working with 2-methyl-1,4-naphthoquinone, a synthetic form of vitamin K (K_3_) that shares similarities with 1,4-NQ, Shang et al. stated that its EC50 on A549 cells is 46.8µM, a value similar to the EC50 reported in the present results [33,34]. Previous studies also suggested that 1,4-NQ is toxic to cells by modulating the metabolic pathways through a combined effect of oxidative stress and energy depletion [33,35,36]. It is also known that 1,4-NQ can interact with glutathione, thus modifying this compound and causing its depletion in cells [34].

Quinones are known to cause microtubule disarrangement and to induce the formation of reactive oxygen species (ROS), damaging the cell membranes and the DNA (clastogenicity); the latter is also damaged by the inhibition, caused by 1,4-NQ, of the human topoimerase IIα enzyme, an enzyme responsible for maintaining the DNA topology [11,33,34,37,38,39]. Complementary to the formation of ROS, quinones are known to have inflammatory potential, by upregulating inflammatory genes such as IL-6, IL-8, TNF-α, Cyp1a1 and HO-1 [40].

To better determine the effects of the different chemicals on cell structure and behavior, we used the Holomonitor microscope. To identify their cellular effects, compounds were tested at concentrations corresponding to their EC50. The cell index produced by RTCA depends on the surface occupied by the cell and their adhesion strength. The surface occupied by cells results from cell number and cell size. The Holomonitor measures different cell parameters, such as surface, perimeter and volume. The fact that the cell index was significantly decreased at the EC50 for 1,4-NQ and OPA, but the area and perimeter of cells were not changed, points out an effect on cell adhesion; both chemicals decreased the optical volume of cells without affecting cell perimeter and area.

The Holomonitor also produces effects on cell behavior, such as motility, motility speed and migration. Firstly, 1,4-NQ and OPA significantly decreased all these parameters while 2-OH-NQ and PA did not alter them. Cell migration is intrinsically connected to the upregulation of the signal transducer and activator of transcription 3 (STAT-3) [41]. Quinones derived from 1,4-NQ are recognized for decreasing the expression levels of this protein [42], which could explain the decrease in all behavioral parameters associated with 1,4-NQ. It is also interesting to note that both 1,4-NQ and OPA induced a decrease in the migration, motility and motility speed.

Results from the literature are divergent on the toxicity of PA. Ema et al. observed that this chemical is not toxic to pregnant rats and their litter, while Kwack & Lee stated that it decreased the viability of testicle cells by 40% at 100 mM, a concentration 10× higher than the one that caused cell death in our experiment [43,44]. The present study is the first to assess the toxic effects of PA on proliferating lung cells, which is of extreme importance, since, as a by-product of naphthalene, it is present in the air in urban areas.

Phthaldialdehyde is used as a common endoscope disinfectant, and there have been reported cases of severe skin and mucous membrane damage of the lip, tongue, pharynx and esophagus areas that were attributed to inadequate washing after the sterilization of a transesophageal echo device with OPA [45,46]. In the present study, OPA decreased the cell index and the optical volume, migration, motility and motility speed when compared to a control. In vivo studies showed that OPA caused fibrosis, hyperplasia, chronic active inflammation and squamous metaplasia in the lung, causing even histiocytic cellular infiltration in the alveolus. However, none of these lesions are known to cause a decrease in the pneumocyte volume or migration parameters; thus, the question regarding the mechanisms that OPA uses to cause these effects remains unanswered.

When comparing RTCA and the Holomonitor, both methods are real-time, without labelling, and useful for assessing the toxicity of all components. However, as RTCA produces an impedance value reflecting cell proliferation, it can be affected by cell surface and adhesion strength. In this manner, the Holomonitor brings a complementary method of determining the impact of a chemical on cells.

## 5. Conclusions

In conclusion, 1,4-NQ is the most toxic by-product of naphthalene, with an EC50 value that is one hundred times higher than those of other compounds, inducing structural and behavioral alterations in A549 cells. Despite their similar EC50 values on RTCA, 2-OH-NQ and PA showed no alteration in cell parameters, while OPA significantly altered optical volume, motility, motility speed and migration. The present study highlights the perfect complementarity of RTCA and the Holomonitor microscope to study alterations induced by chemicals.
